# Amylases in the Human Vagina

**DOI:** 10.1128/mSphere.00943-20

**Published:** 2020-12-09

**Authors:** Kenetta L. Nunn, Geremy C. Clair, Joshua N. Adkins, Kristin Engbrecht, Thomas Fillmore, Larry J. Forney

**Affiliations:** a Institute for Bioinformatics and Evolutionary Studies, University of Idaho, Moscow, Idaho, USA; b Bioinformatics and Computational Biology Graduate Program, University of Idaho, Moscow, Idaho, USA; c Pacific Northwest National Laboratory, Richland, Washington, USA; d Department of Biological Sciences, University of Idaho, Moscow, Idaho, USA; University of Michigan—Ann Arbor

**Keywords:** amylase, glycogen, *Lactobacillus*, vaginal microbiome

## Abstract

Dominance of *Lactobacillus* species in vaginal communities is a hallmark of healthy conditions in the female genital tract. Key nutrients for lactobacilli include sugars produced when glycogen is degraded by α-amylase in the vagina. While α-amylase activity has been demonstrated in vaginal fluids, it is unclear whether α-amylases are produced solely by the host, bacteria in the vagina, or both. We screened cervicovaginal mucus from 23 reproductive-age women, characterized the species composition of vaginal communities, measured vaginal pH, and determined levels of amylase activity, glycogen, and lactic acid. Based on differences in these measured variables, one sample from each of four individual donors was selected for metagenomic and proteomic analyses. Of eight putative bacterial amylases identified in the assembled bacterial metagenomes, we detected four in vaginal fluids. These amylases were produced by various bacteria in different vaginal communities. Moreover, no two communities were the same in terms of which bacteria were producing amylases. Although we detected bacterial amylases in vaginal fluids, there was no clear association between the bacterial species that was dominant in a community and the level of amylase activity. This association was likely masked by the presence of human α-amylase, which was also detected in vaginal fluids. Finally, the levels of amylase activity and glycogen were only weakly associated. Our findings show, for the first time, that multiple amylases from both bacterial and human origins can be present simultaneously in the vagina. This work also suggests that the link between glycogen, amylase, and *Lactobacillus* in the vagina is complex.

**IMPORTANCE** In this study, we show that multiple bacteria in the vaginal community produce amylases that hydrolyze glycogen into simpler sugars (i.e., maltose and maltotriose). These sugars serve as “common goods” that sustain bacterial populations in vaginal communities. Given the temporal changes that are observed in the human vaginal microbiome, we expect the kinds of bacterial amylases produced will also vary over time. These differences influence the pool of resources that are broadly shared and shape the species composition of the vaginal bacterial community.

## Abstract (precis)

In this study, we show that multiple bacteria in the vaginal community produce amylases that hydrolyze glycogen into simpler sugars (i.e., maltose and maltotriose). These sugars serve as “common goods” that sustain bacterial populations in vaginal communities.

## INTRODUCTION

Bacterial communities in the human vagina play an integral role in maintaining women’s genital health. Within these communities, *Lactobacillus* species provide a key ecosystem service by producing lactic acid (1–4), which is thought to restrict pathogenic organisms from colonizing the vagina (3). The abundances of lactobacilli and the overall composition of vaginal microbiota differ markedly between women (5–10). Moreover, some women have vaginal communities that consistently lack appreciable numbers of lactobacilli, whereas others experience periods of time when their communities transition to states where the abundance of lactobacilli is low (11–14). All instances in which lactobacilli are in low abundance constitute windows of elevated risk for disease and adverse reproductive outcomes (reviewed in reference 15). Thus far, the key drivers of vaginal community composition are poorly understood.

The abundance of *Lactobacillus* spp. in the vagina is positively associated with the levels of estrogen and vaginal glycogen content (16, 17). Increased estrogen levels cause the vaginal epithelium to thicken, thereby prompting the accumulation of glycogen within epithelial cells. These events correlate with increases in the absolute abundances of bacteria and the proportions of *Lactobacillus* spp. in the vagina. In ways that are not fully understood, estrogen and glycogen are thought to create an environment that stimulates the proliferation of vaginal lactobacilli, possibly because sugars derived from glycogen serve as a source of carbon (18).

Glycogen is a regular repeating glucose polymer, linearly connected by α-1,4-glycosidic bonds with branching at roughly every 8 to 10 residues by way of α-1,6-glycosidic bonds (reviewed in reference 19). α-Amylase is an extracellular glycoside hydrolase that cleaves α-1,4-glycosidic bonds, producing maltose, maltotriose, and α-limit dextrins (20). This enzyme, which is presumed to be host derived (21, 22), is likely required to first depolymerize glycogen to form simpler sugars that can be transported into cells where they are further catabolized by *Lactobacillus* to produce lactic acid (21). Humans harbor two α-amylase isozymes, which are thought to be expressed only in the salivary glands (AMY1) and the pancreas (AMY2A and AMY2B) (23, 24). Meanwhile, many microbial species have been shown to produce α-amylase in various habitats as well as other kinds of amylases, including but not limited to β-amylase, glucoamylase, α-glucosidase, and pullulanase (25–27). Currently, it is not known whether bacterial species that reside in the human vagina produce amylases.

α-Amylases and other glycoside hydrolases are grouped into various protein families (28) based on differences in substrate specificity and three-dimensional structure. The largest glycoside hydrolase family, glycoside hydrolase 13 (GH13), is colloquially known as the α-amylase family. GH13 includes numerous types of hydrolases that have been further divided into at least 35 subfamilies of proteins (28, 29). Given this heterogeneity, we hypothesize there are multiple amylases present from different sources in the human vagina, and the greatest diversity is produced by bacteria. To explore this, we obtained self-collected cervicovaginal mucus (CVM) samples from 23 reproductive-age women. We measured vaginal pH in addition to the levels of amylase activity, glycogen, and lactic acid and determined which bacterial species were present in these communities. We chose a subset of four women based on differences in these measurements and vaginal microbiota and analyzed the metagenomes of these vaginal communities to identify putative amylase enzymes. Furthermore, we used proteomics to determine which of these putative amylase enzymes were present in CVM. We detected both host and bacterial amylases in vaginal fluids with the ability to hydrolyze both α-1,4- and α-1,6-glycosidic bonds in glycogen. Our findings suggest that multiple amylase types and sources are typically present in the human vagina.

## RESULTS

### Study cohort.

A summary of the metadata related to study participants is shown in Table S1 in the supplemental material. Study participants ranged from 19 to 45 years of age, with an average age of 27. Women reported that they were in good health, were not pregnant, had their ovaries and uterus, and had not had vaginal intercourse in the 48 h before sample collection. The species compositions of vaginal communities in this cohort of 23 women were similar to those found in other studies of healthy reproductive-age women (see Table S2) (10). In contrast, vaginal pH, total protein, amylase activity, and levels of glycogen and lactic acid varied substantially (see Table S3).

10.1128/mSphere.00943-20.2 TABLE S1 Metadata for women in the study cohort. Download Table S1, XLSX file, 0.1 MB. Copyright © 2020 Nunn et al. 2020 Nunn et al. This content is distributed under the terms of the Creative Commons Attribution 4.0 International license.

10.1128/mSphere.00943-20.3 TABLE S2 Relative proportions of bacteria in each community. Download Table S2, PDF file, 0.1 MB. Copyright © 2020 Nunn et al. 2020 Nunn et al. This content is distributed under the terms of the Creative Commons Attribution 4.0 International license.

10.1128/mSphere.00943-20.4 TABLE S3 Attributes of CVM samples collected from reproductive age women. Download Table S3, PDF file, 0.1 MB. Copyright © 2020 Nunn et al. 2020 Nunn et al. This content is distributed under the terms of the Creative Commons Attribution 4.0 International license.

The samples collected in this study were used to characterize bacterial community compositions and the relationship between amylase activity and glycogen levels in the vagina. However, we selected only four CVM samples from donors F02, F06, F08, and F12 for additional analyses to assemble the bacterial metagenomes and identify the *in vivo* expression of amylase proteins using proteomics. The corresponding metadata for these samples are shown in Table S4. This subset was chosen based on whether samples were below, at, or above the mean value for vaginal pH, amylase activity, and glycogen and lactic acid levels. We also factored in differences in vaginal community composition.

10.1128/mSphere.00943-20.5 TABLE S4 Characteristics of samples selected for shotgun metagenomics and proteome analysis. Download Table S4, PDF file, 0.1 MB. Copyright © 2020 Nunn et al. 2020 Nunn et al. This content is distributed under the terms of the Creative Commons Attribution 4.0 International license.

### Glycogen levels are negatively correlated with amylase activity.

**FIG 1:**
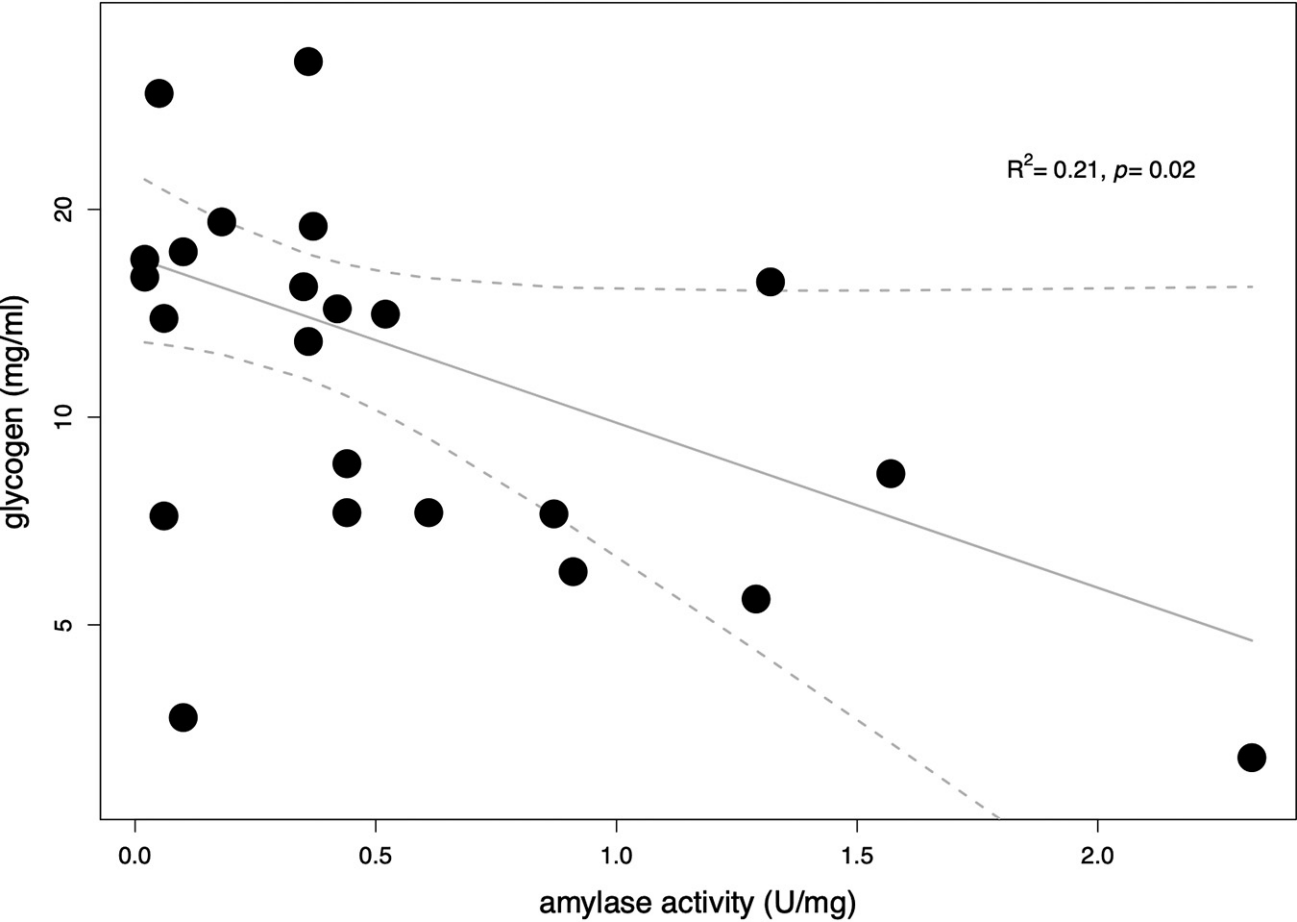
Scatterplot of glycogen measurements (milligrams per milliliter) as a function of amylase activity (units per milligram of total protein). The points in the graph represent glycogen measurements for each sample, plotted with the corresponding amylase activity. The regression line was generated by fitting a generalized linear model to these data points. The dashed lines represent the 95% confidence interval for the model fit. The pseudo-*R*^2^ and the *P* value for the model are shown at the top right.

Given that amylase depolymerizes glycogen into simpler sugars that can be metabolized by bacteria in the vaginal community (21), we expected there to be a negative association between amylase activity and vaginal glycogen levels. To evaluate this relationship, we measured amylase activity and glycogen levels in CVM samples and fit a generalized linear model to these data by using a log link function. The resulting scatterplot of glycogen as the response variable and amylase activity as the predictor variable is shown in Fig. 1. The model indicated there was a negative relationship between amylase activity and glycogen levels (*R*^2^ = 0.21, *P* = 0.02), which implies that glycogen levels tend to decrease with increasing amylase activity in the vagina. However, it should be noted that there was considerable variability in glycogen levels (range, 3.7 to 32.8 mg/ml) when the corresponding amylase activity was less than 0.5 U/mg total protein.

### Amylase activity is not associated with bacterial species composition in the vagina.

**FIG 2:**
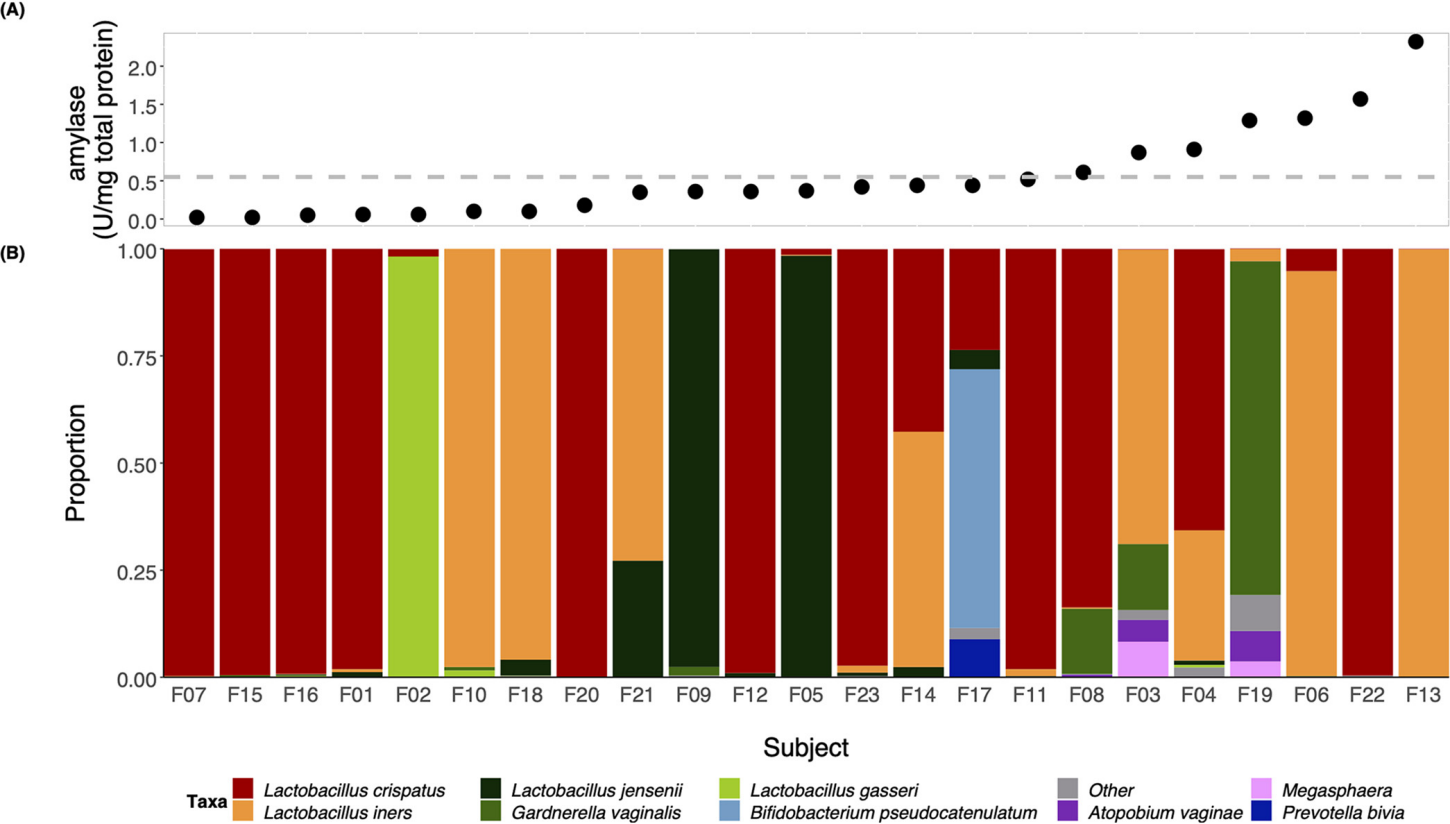
Amylase activity relative to bacterial species composition in the human vagina. Amylase activity (units per milligram total protein) in each sample was arranged in increasing order (A) along with the corresponding stacked bar (B). The dashed gray line in panel A represents the mean of amylase activity across samples (0.55 U/mg). The stacked bars represent the relative proportions of bacteria in each community. The colors for bacterial taxa are shown in the legend at the bottom.

To get a sense as to whether amylase activity was associated with the composition of vaginal communities, we prepared a summary plot of bacterial species composition for each sample with the corresponding amylase activity measurement (Fig. 2). Based on the 16S rRNA gene sequence data, 21 of the 23 women studied had communities dominated by species of *Lactobacillus*, one woman had a community dominated by Gardnerella vaginalis, and the other woman had a community dominated by Bifidobacterium pseudocatenulatum. The levels of amylase varied widely between communities that were similar in composition. Thus, there was no clear association between the bacterial species that dominated a community and the level of amylase activity present. Given this, we could not reach any conclusions as to whether the presence of any particular bacterium was related to the amylase activity detected in the vagina.

### The electrophoretic mobility of amylase proteins varies across donors.

Native polyacrylamide gel electrophoresis (PAGE) has been widely used to resolve amylase proteins and can distinguish between the two forms of human α-amylase (reviewed in reference 24). Thus, we applied this technique to determine if we could detect and differentiate amylases in CVM samples. We detected amylase activity in at least 18 of the CVM samples (see Fig. S1). In each of those samples, including the controls, there were multiple protein bands present. Furthermore, the electrophoretic mobility of the protein bands was quite variable across samples (Fig. S1). Given that there were multiple bands observed in purified controls, it is unclear whether the bands seen in one sample reflect multiple kinds of amylase proteins or are the result of posttranslational modifications that yield multiple forms of a single amylase protein (30). We also found that the zones of starch hydrolysis, which is reflected by the dark banding pattern in the image, varied in intensity. This variability could be the result of several factors, such as differences in the levels and activity of amylase proteins or differences in substrate specificity, gene expression, and protein stability, among others.

10.1128/mSphere.00943-20.7 FIG S1 Amylase enzymes in vaginal fluids collected from reproductive-age women separated by native PAGE. In this figure are all of the images obtained from native PAGE gels that were incubated in 1% starch and stained with iodine solution after separating amylase proteins in CVM from all of the women in our study. These images were inverted so that the dark background shows up white and the clear zones indicating hydrolysis of starch show up dark. Thus, the dark bands reflect amylase isozymes that were resolved in each donor sample. The donor identifier (ID) for each sample is below the gel image. A total of 20 μg of total protein was loaded for each sample. PA (human pancreatic α-amylase) and GA (*Rhizopus spp.* glucoamylase) are two commercial amylases that were used as positive controls for the assay. Download FIG S1, PDF file, 0.1 MB. Copyright © 2020 Nunn et al. 2020 Nunn et al. This content is distributed under the terms of the Creative Commons Attribution 4.0 International license.

**FIG 3:**
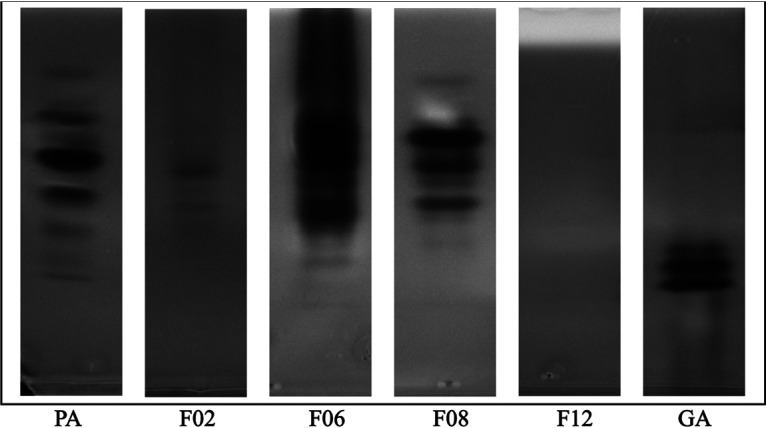
Amylase enzymes in vaginal fluids collected from donors F02, F06, F08, and F12 separated by native PAGE. These columns represent image slices taken from Fig. S1 in the supplemental material (containing all of the images of native PAGE gels completed for all samples) for donor samples F02, F06, F08, and F12. A total of 20 μg of total protein was loaded for each sample. After the amylase proteins were separated in the gels, gels were incubated in 1% starch to enable starch hydrolysis and stained with iodine solution to observe where hydrolysis took place. The images that the slices were extracted from were inverted so that the dark background shows up white and the clear zones indicating hydrolysis of starch show up dark. Thus, the dark bands reflect amylase isozymes that were resolved in each donor sample. We were unable to resolve any amylase proteins in donor F12. PA (human pancreatic α-amylase) and GA (*Rhizopus* spp. glucoamylase) are two commercial amylases that were used as positive controls for the assay.

In Fig. 3, we highlight the gel results from the four samples that were selected to analyze using metagenomics and proteomics: F02, F06, F08, and F12. There were two protein bands in CVM from donor F02, six were visible in F06, and seven were detected in F08. For unknown reasons, we did not observe amylase protein bands in CVM from F12 or four other CVM samples (Fig. S1). However, we were able to measure amylase activity in all of the samples.

### Several putative amylase proteins are present in vaginal bacterial metagenomes.

In this study, we sought to identify amylase proteins produced by bacteria in human vaginal communities. To do this, we first needed to recover the bacterial genomes that were present in the CVM samples collected. Therefore, we performed shotgun metagenomics on DNA obtained from donor samples F02, F06, F08, and F12. We assembled sequence reads into contigs and binned those contigs into metagenome-assembled genomes (MAGs). To identify the taxa present in our samples, MAGs were classified to the species level using the GTDB taxonomy database (31). We recovered two MAGs from the metagenome of donor F02 and found they were derived from Lactobacillus gasseri and *Bifidobacterium vaginale* (Gardnerella vaginalis). One MAG was recovered from F06 and identified as Lactobacillus iners. Five MAGs were recovered from F08 and identified as Fannyhessea vaginae, Ureaplasma parvum, Lactobacillus crispatus, *Bifidobacterium vaginale*, and *Bifidobacterium lacrimalis* (Peptoniphilus lacrimalis). Finally, three MAGs were recovered from F12 and identified as Lactobacillus crispatus, Lactobacillus jensenii, and *Bifidobacterium vaginale*.

Next, we annotated our recovered MAGs to identify putative amylase genes and link the amylases with their bacterial hosts. We found a total of eight different GH13 hydrolase proteins that were homologous to known hydrolase proteins within the GH13 protein family (EC number 3.2.1.−). All of these proteins can cleave either α-1,4- or α-1,6-glycosidic linkages, and some could hydrolyze both (Table 1). Moreover, the proteins we recovered were detected in multiple MAGs. The eight different proteins were annotated as α-amylase (EC 3.2.1.1), glucan 1,6-α-glucosidase (EC 3.2.1.70), oligo-1,6-glucosidase (3.2.1.10), intracellular maltogenic amylase (EC 3.2.1.−), trehalose synthase/amylase TreS (EC 3.2.1.1), glycogen operon protein glgX (EC 3.2.1.−), pullulanase (EC 3.2.1.41), and neopullulanase (EC 3.2.1.135).

**TABLE 1:** Putative amylase proteins annotated in the metagenomic assembled genomes of bacteria in human vaginal samples

Sample	Taxon	Protein	EC no.	Reaction	Gene(s)
F02	*Bifidobacterium vaginale*^*a*^	Oligo-1,6-glucosidase	3.2.1.10	α-1,6-Glycosidic linkages	*malL*
		Pullulanase	3.2.1.41	α-1,6-Glycosidic linkages	*pulA*
		Trehalose synthase/amylase TreS	3.2.1.1	α-1,4-Glycosidic linkages	*treS*
		α-Amylase	3.2.1.1	α-1,4-Glycosidic linkages	*aml*
		Neopullulanase	3.2.135	α-1,4- and α-1,6-Glycosidic linkages	*nplT*
	Lactobacillus gasseri	Glucan 1,6-α-glucosidase	3.2.1.70	α-1,6-Glycosidic linkages	*dexB*
		Intracellular maltogenic amylase	3.2.1.−	α-1,4-Glycosidic linkages	*bbmA*
		Oligo-1,6-glucosidase	3.2.1.10	α-1,6-Glycosidic linkages	*malL*
F06	Lactobacillus iners	Glucan 1,6-α-glucosidase	3.2.1.70	α-1,6-Glycosidic linkages	*dexB*
		Intracellular maltogenic amylase	3.2.1.−	α-1,4-Glycosidic linkages	*bbmA*
		Oligo-1,6-glucosidase	3.2.1.10	α-1,6-Glycosidic linkages	*malL*
		Pullulanase	3.2.1.41	α-1,6-Glycosidic linkages	*amyX_1*, *amyX_2*, *amyX_3*
F08	*Bifidobacterium lacrimalis*^*b*^	Glycogen operon protein GlgX	3.2.1.−	α-1,6-Glycosidic linkages	*glgX*
		Oligo-1,6-glucosidase	3.2.1.10	α-1,6-Glycosidic linkages	*malL_2*
		Neopullulanase 2	3.2.135	α-1,4- and α-1,6-Glycosidic linkages	*tvall*
		Oligo-1,6-glucosidase 1	3.2.1.10	α-1,6-Glycosidic linkages	*malL_1*
		Pullulanase	3.2.1.41	α-1,6-Glycosidic linkages	*amyX*, *pulA*
	*Bifidobacterium vaginale*	Neopullulanase	3.2.135	α-1,4-, and α-1,6-Glycosidic linkages	*nplT*
		Oligo-1,6-glucosidase	3.2.1.10	α-1,6-Glycosidic linkages	*malL*
		Pullulanase	3.2.1.41	α-1,6-Glycosidic linkages	*pulA*
		Trehalose synthase/amylase TreS	3.2.1.1	α-1,4-Glycosidic linkages	*treS*
	Lactobacillus crispatus	Glucan 1,6-α-glucosidase	3.2.1.70	α-1,6-Glycosidic linkages	*dexB*
		Intracellular maltogenic amylase	3.2.1.−	α-1,4-Glycosidic linkages	*bbmA*
		Oligo-1,6-glucosidase	3.2.1.10	α-1,6-Glycosidic linkages	*malL_1*, *malX_2*
		Pullulanase	3.2.1.41	α-1,6-Glycosidic linkages	*amyX*
F12	*Bifidobacterium vaginale*	Glycogen operon protein GlgX	3.2.1.−	α-1,6-Glycosidic linkages	*glgX*
		Neopullulanase	3.2.135	α-1,4- and α-1,6-Glycosidic linkages	*nplT*
		Oligo-1,6-glucosidase	3.2.1.10	α-1,6-Glycosidic linkages	*malL*
		Pullulanase	3.2.1.41	α-1,6-Glycosidic linkages	*pulA*
		Trehalose synthase/amylase TreS	3.2.1.1	α-1,4-Glycosidic linkages	*treS*
	Lactobacillus crispatus	Glucan 1,6-α-glucosidase	3.2.1.70	α-1,6-Glycosidic linkages	*dexB*
		Intracellular maltogenic amylase	3.2.1.−	α-1,6-Glycosidic linkages	*bbmA*
		Oligo-1,6-glucosidase	3.2.1.10	α-1,6-Glycosidic linkages	*malL*
		Pullulanase	3.2.1.41	α-1,6-Glycosidic linkages	*amyX*
	Lactobacillus jensenii	Glucan 1,6-α-glucosidase	3.2.1.70	α-1,6-Glycosidic linkages	*dexB*
		Intracellular maltogenic amylase	3.2.1.−	α-1,6-Glycosidic linkages	*bbmA*
		Oligo-1,6-glucosidase	3.2.1.10	α-1,6-Glycosidic linkages	*malL*

a *Bifidobacterium vaginale* is referenced from the GTDBTK taxonomy database and reported here but listed as Gardnerella vaginalis in the NCBI taxonomy database.

b *Bifidobacterium lacrimalis* is referenced from the GTDBTK taxonomy database and reported here but listed as Peptoniphilus lacrimalis in the NCBI taxonomy database.

### Vaginal amylases are produced by both the host and bacteria in the vaginal community.

The data from amylase assays, native PAGE gels, and metagenome sequence analysis collectively suggest that multiple amylases are produced by bacteria in the vagina. To determine which of the putative amylases were expressed *in vivo*, we surveyed the proteomes of CVM samples from donors F02, F06, F08, and F12. Proteins were extracted from CVM samples and analyzed using liquid chromatography-tandem mass spectrometry (LC-MS/MS). Furthermore, we took the sequences of the proteins that were expressed and performed a search against the protein annotations from our recovered MAGs and the human proteome to identify the amylase proteins and their source. For each donor, the proteome profiles were generated, at least in duplicates. Hierarchical clustering of all of the proteins detected showed that the women fell into their own distinct groups (see Fig. S2). Among the proteomes of the four samples, a total of 4,426 proteins were quantified and were partitioned into 1,092 bacterial proteins and 3,334 human proteins.

10.1128/mSphere.00943-20.8 FIG S2 Dendrogram depicting the clustering of the proteomes of the samples originating from different donors. All of the proteins detected were included in the clustering analysis. Samples clustered using Ward’s method on Euclidean distances calculated from the protein abundances. F02 is highlighted in orange, F06 is highlighted in blue, F08 is highlighted in yellow, and F12 is highlighted in purple. Download FIG S2, PDF file, 0.1 MB. Copyright © 2020 Nunn et al. 2020 Nunn et al. This content is distributed under the terms of the Creative Commons Attribution 4.0 International license.

**FIG 4:**
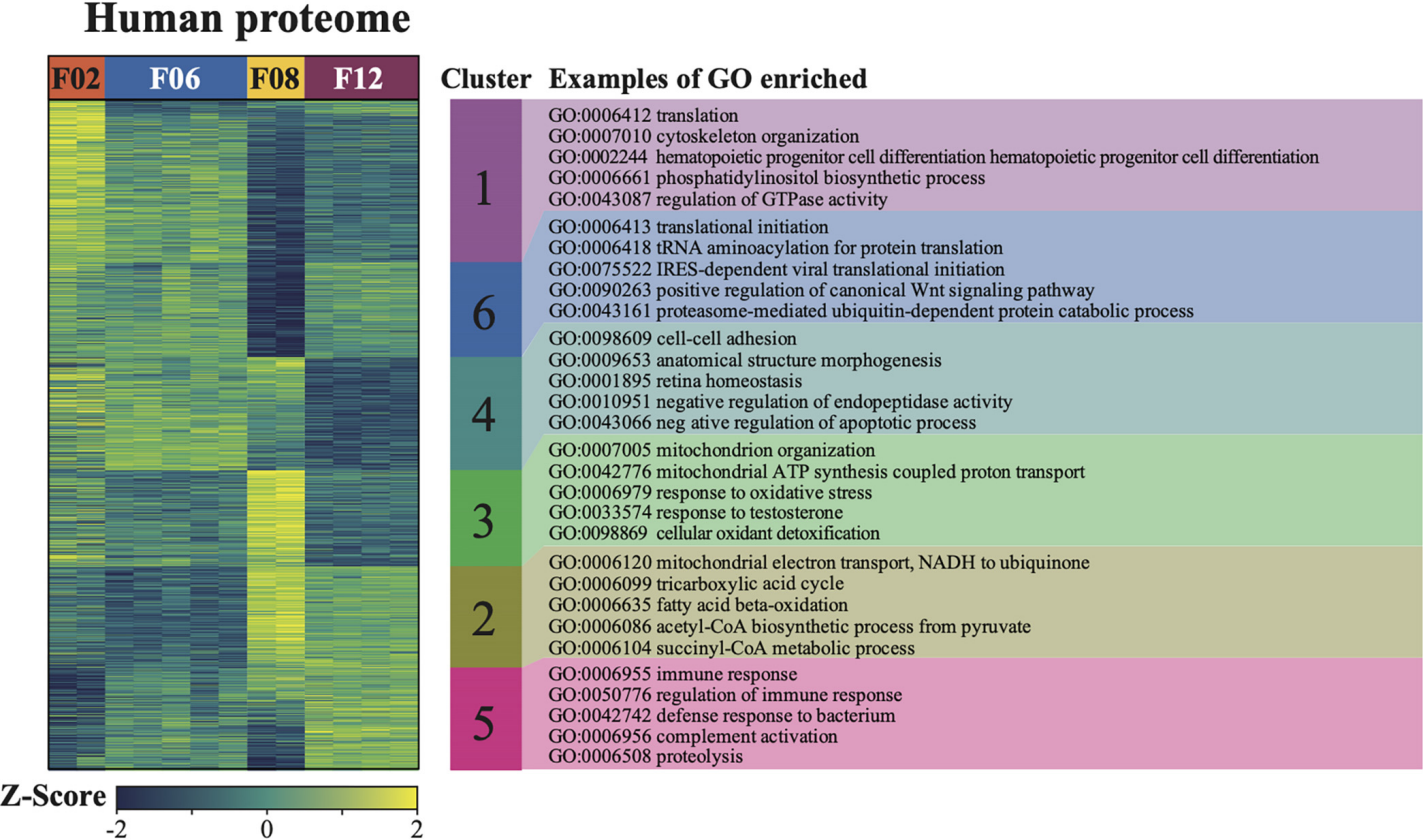
Human proteins expressed in vaginal fluids of donors F02, F06, F08, and F12. (Left) Heat map that reflects the relative abundances of human proteins detected in each sample. Relative abundances are indicated by the Z-scores as shown at the bottom. Blue values indicate low abundances based on the mean of all of the proteins in a sample, whereas yellow indicates high abundances. (Right) Clusters identified in the enrichment analysis that identified GO terms that were enriched in the proteome for each sample.

For the human proteome specifically, women were vastly different in the abundances of various proteins and their functions (Fig. 4). An analysis of variance (ANOVA) confirmed that 39.1% of the human proteins identified were significantly different in their abundances among the four CVM samples (*N* = 1,304; *P* < 0.05). To determine the potential functional differences between the proteomes of the four women, we performed an enrichment analysis on the clusters that were obtained. Figure 4 highlights the Gene Ontology (GO) terms that were enriched in the proteomes of each woman (Fisher’s exact *P* < 0.05). For example, cluster 3 represents the proteins that were higher in abundance in donor F08 and was characterized by proteins involved in mitochondrial ATP synthesis (e.g., GO:0007005, GO:0042776, GO:0006123, GO:0005743, and GO:0005739) and response to oxidative stress (e.g., GO:0006979, GO:0098869, and GO:0042744). Meanwhile, cluster 6 comprised proteins that were higher in abundance in donor F12, consisting of proteins involved in the host immune response in defense against bacteria (e.g., GO:0006955, GO:0050776, GO:0042742, GO:0019731, GO:0050900, and GO:0002576). A detailed list of enriched GO terms is provided in the Table S5.

10.1128/mSphere.00943-20.6 TABLE S5 Proteomic data (includes global statistics, microbiome proteome, detected amylases of the family GH13, and human proteome). Download Table S5, XLSX file, 8.7 MB. Copyright © 2020 Nunn et al. 2020 Nunn et al. This content is distributed under the terms of the Creative Commons Attribution 4.0 International license.

**FIG 5:**
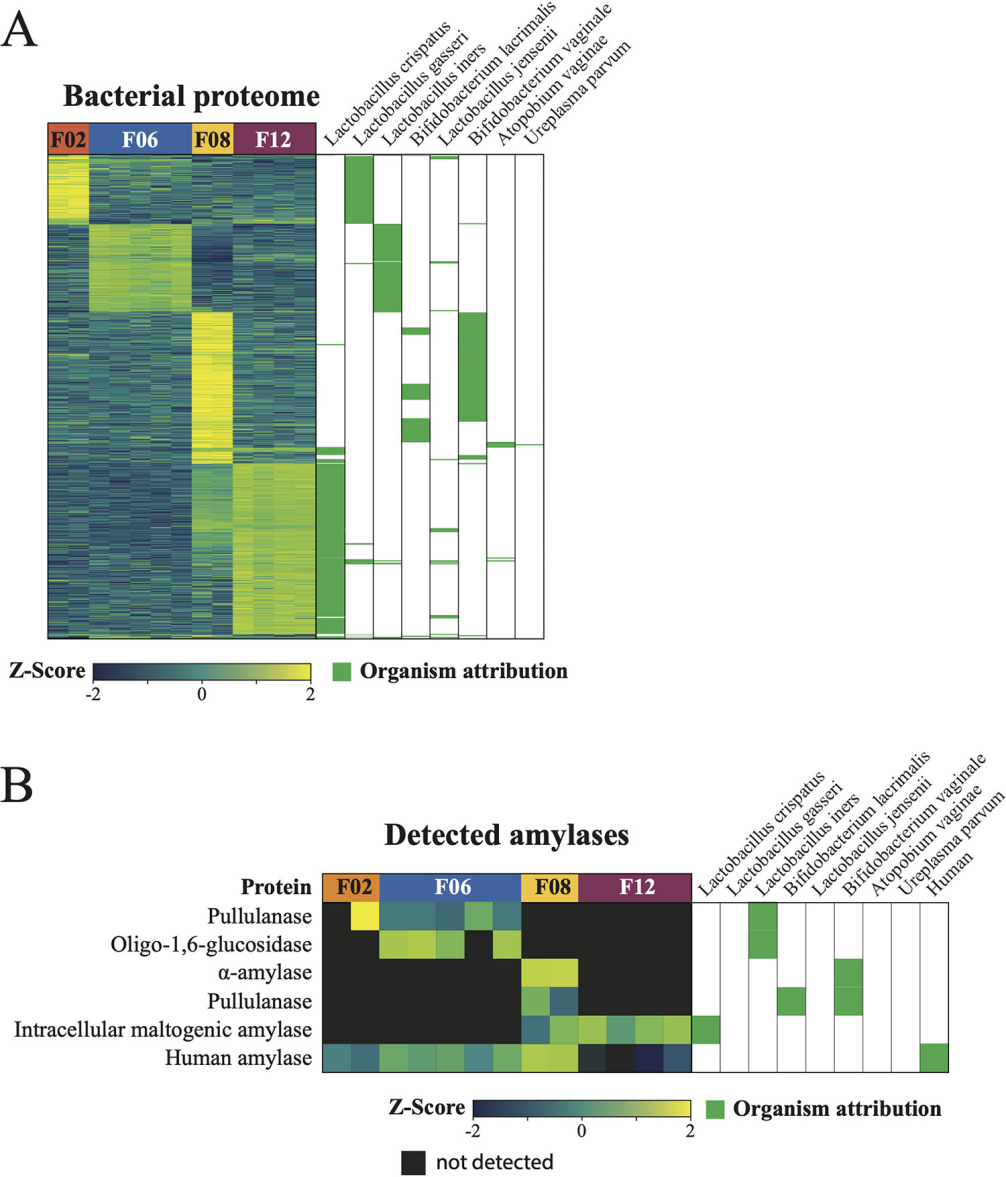
Bacterial proteins expressed in vaginal fluids from donors F02, F06, F08, and F12. (A) Heat map that reflects the relative abundances of bacterial proteins detected in each sample. Relative abundances are indicated by the Z-scores shown at the bottom. (Right) Green boxes indicate in which MAG (bacteria) the proteins were detected. (B) Putative bacterial and human amylases that were detected in vaginal fluids using LC-MS/MS. The relative abundance of the protein (listed on the left) is shown in individual heat maps below each sample. Relative abundances are indicated by the Z-scores shown at the bottom. Black squares indicate that the protein was not detected in a sample. Similar to that in panel A, the green boxes at the right show which MAG the protein sequence was found in.

The bacterial proteomes for donors F02, F06, and F12 (Fig. 5A) were enriched with proteins that were derived from the dominant bacteria in their respective vaginal communities, based on the 16S rRNA gene proportion data (Fig. 2). Specifically, proteins from L. gasseri were highly abundant in F02. Meanwhile, proteins from L. crispatus dominated the proteome of F12. *L. iners* was the dominant species in donor F06 and the only MAG identified in this donor; as expected, the protein abundances reflected this. For donor F08, the majority of the enriched proteins were attributed to *B. vaginale* and *B. lacrimalis*; only some were attributed to L. crispatus, which was the dominant species based on the proportions of 16S rRNA gene profiles (Fig. 5A). Nevertheless, the overall number of detected proteins attributed in F08 to L. crispatus (286 proteins) was higher than the number of proteins attributed to *B. vaginale* (263 detected proteins) and to *B. lacrimalis* (118 proteins) (Table S5). These results suggest that while the overall abundance of L. crispatus was the highest, other species may have been more metabolically active according to their protein abundances. These stark differences in the secreted human proteins and the bacterial proteomes among the four women suggest that the microbiota might shape the profile of the human secreted proteins in CVM. However, a study that includes a much larger number of donors would be necessary to gain a better understanding of these relationships.

Focusing specifically on amylase proteins, we identified four amylases in the proteomes of our samples that matched translated protein sequences annotated in the corresponding bacterial metagenomes (Fig. 5B). These proteins included an α-amylase from *B. vaginale* (F08), an intracellular maltogenic amylase from L. crispatus (F08 and F12), an oligo-1,6-glucosidase from *L. iners* (F06), and pullulanase from *L. iners* (F06), *B*. *vaginale* (F08), and *B. lacrimalis* (F08). There was also a pullulanase detected in one of the replicates from F02. However, this pullulanase did not map to either of the MAGs recovered from F02. A protein group composed of three human α-amylases was also identified in this data set at various levels in all samples. Within this group, 23 peptides were mapped to AMY1_HUMAN (AMY1, salivary type), 18 peptides were mapped to AMYP_HUMAN (AMY2A, pancreatic type), and 20 peptides were mapped to AMY2B_HUMAN (AMY2B, pancreatic type). Normalized protein abundances were higher for the bacterial amylases than for human amylases detected in CVM from donors F02, F06, and F12. The opposite was true for donor F08.

## DISCUSSION

Alpha-amylase is thought to be a key enzyme in the vaginal ecosystem that depolymerizes glycogen into simpler oligosaccharides, and these sugars are presumed to be fermented by *Lactobacillus* to produce lactic acid (15, 21, 32, 33). Previously, researchers have measured amylase activity in vaginal fluids (21, 22, 34) and examined the expression of amylases in other genital tract tissues (35, 36). However, to our knowledge, this is the first study to demonstrate bacterial amylases in the vagina. We found five different putative amylases within the GH13 α-amylase protein family in vaginal fluids from a subset of women in our cohort. The four bacterial amylases matched protein annotations from the assembled metagenomes of L. crispatus, *L. iners*, *B. lacrimalis*, and *B. vaginale*. The fifth amylase that we detected was human α-amylase.

Although we detected bacterial amylases in vaginal fluids, there was no clear association between the levels of amylase and the dominant species in the vaginal community. We propose two possible explanations for this result. First, the amylases detected were produced by multiple members of the bacterial community. Second, our proteomics analysis detected a combination of bacterial and human amylases in the four women analyzed. Taken together, these factors likely masked any association we might have observed between bacterial species in the vagina and the levels of specific amylases produced. Nasioudis et al. reported that α-amylase activity was lower in women with bacterial vaginosis (BV), a condition in which *Lactobacillus* abundances are low compared to those in women without BV (22). Only two women in our cohort had communities that were not dominated by species of *Lactobacillus*. The levels of amylase were not different between those women and women with *Lactobacillus*-dominant vaginal communities. While the women in our cohort reported that they were not experiencing any odor, itching, or discharge, we did not investigate whether these women had symptomatic or asymptomatic BV.

We found that the profile of bacterial amylases in the vagina differed between women. Furthermore, the amylase proteins detected in vaginal fluids can hydrolyze either α-1,4- or α-1,6-glycosidic bonds. α-Amylase and intracellular maltogenic amylase hydrolyze α-1,4-glycosidic bonds, suggesting that they can hydrolyze linear glucose polymers. Meanwhile, oligo-1,6-glucosidase and pullulanase hydrolyze α-1,6-glycosidic bonds, which provides a means to hydrolyze branched glucose linkages. In the CVM of each donor, except F12, there were amylases that could perform both reactions. This suggests that within a vaginal community, multiple enzymes might be employed to metabolize glycogen by first cleaving branched glucose chains and then hydrolyzing linear glucose monomers. In F12, we only detected amylases that break linear glucose chains, which included intracellular maltogenic amylase from L. crispatus and human α-amylase. Interestingly, F12 had two to four times the amount of glycogen measured in samples F02, F06, and F08. Perhaps this can be attributed to the inability to completely break down glycogen due to the lack of an enzyme that can hydrolyze branched glucose chains in glycogen. To provide a mechanistic understanding of the conditions that are required to depolymerize glycogen *in vivo*, future studies might compare the levels of glycogen in the vagina with the presence or absence of amylase proteins that hydrolyze α-1,4- and α-1,6-glycosidic bonds. Interestingly, α-amylase and glycogen were only weakly associated in the samples collected in our study. There was also quite a bit of variability in glycogen levels when the corresponding amylase activity was <0.5 U/mg protein. These observations could have resulted from the presence of multiple amylases in the vagina that differ in their substrate specificity and catalytic rate constants. All of this indicates that the relationship between the vaginal community, α-amylase, and glycogen is much more complex than previously thought.

The question still remains whether *Lactobacillus* spp. preferentially metabolize glycogen or simpler sugars in the vagina. Until recently, studies indicated that *Lactobacillus* cannot metabolize glycogen, because lactobacilli could not grow on media containing glycogen as the sole carbon source in the laboratory (21, 37–39). For example, van der Veer et al. cultured 25 L. crispatus vaginal isolates in medium supplemented with glycogen (27). They found that one of those isolates showed no growth at all, while six isolates grew “less efficiently” on glycogen. The authors compared the genomes of the isolates and attributed the lack of or reduced growth of those seven isolates to an N-terminal deletion in a pullulanase type I gene (27). These results did not include expression analyses of the pullulanase type I gene in the growth medium from the vaginal isolates. Others have also identified genes with homology to pullulanase in vaginal metagenomes, but direct evidence that they are expressed is lacking (40). In our study, we did not detect pullulanase from L. crispatus in the proteins identified in vaginal fluids. However, we did find a pullulanase produced by *L. iners*. We also found two other *Lactobacillus* amylases in vaginal fluids: oligo-1,6-glucosidase from *L. iners* and intracellular maltogenic amylase from L. crispatus. Whether these amylases are sufficient for vaginal lactobacilli to metabolize glycogen *in vivo* remains unknown.

Previous studies have detected amylase genes in vaginal microbes (25, 41, 42), but until now, it has not been confirmed whether these proteins are actually expressed in the vagina. While we detected multiple amylases in the vaginas of four women, we did not determine whether they actually break down glycogen. Future studies might aim to biochemically characterize these enzymes to understand their substrate specificities, the factors that regulate their expression, and how the metabolic products (oligosaccharides, maltose, and glucose) are partitioned among community members.

## MATERIALS AND METHODS

### Study design.

Reproductive-age women were recruited to participate in a pilot study designed to learn more about the amylases present in the human vagina. This study was approved by the Institutional Review Board at the University of Idaho (IRB number 18-118). Informed written consent was obtained from each study participant before sample collection. Women were enrolled if they were not pregnant, were not experiencing vaginal bleeding, did not have an intrauterine device, were not taking oral or topical antibiotics to treat vaginal infections, and were not experiencing vaginal symptoms that might indicate the presence of a sexually transmitted infection or disease. Moreover, participants were asked to refrain from having vaginal intercourse and using vaginal lubricants within 48 h of sample collection, as those events could influence the vaginal microbiome (11, 43, 44). Donors were given a unique identifier starting with the letter F followed by a number, and these designations are used throughout this work.

Self-collected vaginal samples were obtained from study participants, as previously described (45). Briefly, participants were provided a 50-ml conical tube and a commercially available device used to collect menstrual fluid called the Softcup. Participants were asked to insert the Softcup into their vagina, leave it there for 1 min, remove it, and then place it into the 50-ml conical tube, which was placed on ice until further processing. Cervicovaginal mucus (CVM) was collected at the bottom of the tube by centrifugation at 2,000 rpm for 5 min and then transferred to a 1.5-ml tube. The volume, weight, and pH of the samples were recorded in addition to observations on sample consistency and appearance. To measure pH, 10 μl of mucus was spread over a commercially available pH stick (EMD Millipore), and based on the color change, pH was recorded in increments of 0.3. Samples were then aliquoted into 1.5-ml tubes and stored at −80°C.

### Amylase activity measurements and resolution by native PAGE.

CVM samples were centrifuged at 14,000 rpm for 5 min to separate the mucus into solid and aqueous phases. The aqueous phase was aspirated, transferred to a clean 0.5-ml tube, and stored at −20°C until further use. Amylase activity in CVM was measured using the commercial EnzChek Ultra amylase assay kit (Molecular Probes) according to the manufacturer’s instructions. *Bacillus* sp. α-amylase (A-6380; Sigma) was used to generate a standard curve. Duplicate measurements of amylase activity were recorded in units per milliliter. These measurements were normalized by total protein in samples, measured using the Bradford assay, and recorded as units per microgram of total protein.

We resolved amylase proteins using the aqueous phase of samples prepared as described above by native PAGE. Briefly, 20 μg of total protein was diluted in native sample buffer (Bio-Rad) and loaded on 12% Criterion TGX precast midi protein gels (Bio-Rad). Human pancreatic α-amylase and *Rhizopus* spp. glucoamylase were loaded with samples to serve as positive controls. PAGE was performed in 1× Tris-glycine buffer, and gels received 36 V for 1 h to allow samples to settle into the stacking portion of the gel and then approximately 150 V for 4.5 h. Gels were washed in deionized water for 2 min and incubated for 1 h at 37°C in a 1% starch solution in 0.02 M Tris-Cl with 1 mM CaCl_2_, pH 7.4. Afterward, gels were washed in deionized water for 2 min, incubated in 0.02 M Tris-Cl (pH 7.4) for 10 min at 37°C, and rinsed in deionized water again. Gels were developed by overlaying Miracloth (Calbiochem) soaked previously in 10 mM iodine-14 mM potassium iodine on the gel for 10 min and destained with deionized water. Then, gels were fixed in 1% acetic acid and imaged on the Bio-Rad Gel Doc XR^+^. Iodine stains portions of the gel with intact starch dark blue, leaving segments of the gel in which starch has been hydrolyzed clear. Thus, this method allows for determining whether amylase is present in a sample and visualizing the pattern of protein bands produced.

### Glycogen and lactic acid measurements.

CVM samples were diluted 5-fold (wt/wt) in 1× phosphate-buffered saline (PBS), homogenized by vortexing for 1 min, and centrifuged at 14,000 rpm for 5 min. The supernatant was transferred to a clean 1.5-ml tube and stored at −20°C until further use. Glycogen was quantified using the EnzyChrom glycogen assay kit (BioAssay Systems) according to the manufacturer’s instructions. Duplicate measurements for glycogen were recorded in milligrams per milliliter. To measure the concentrations of lactic acid in the samples, we used commercially available d- and l-lactic acid kits (Bioassays) according to the manufacturer’s protocol. Duplicate measurements of d-, l-, and total lactic acid were recorded in millimolar.

### Bacterial community analysis.

The species composition of vaginal microbial communities was determined by classifying partial 16S rRNA gene sequences (see Text S1). We used methods that were previously described (46) with a modification in the sample volume. In brief, total genomic DNA was extracted from 25 μl of CVM using chemical and mechanical lysis and purified using QIAamp DNA minikits (Qiagen). Genomic DNA concentrations were determined using the Quant-iT PicoGreen double-stranded DNA (dsDNA) assay kit (Invitrogen). For amplicon sequencing, the V1-V3 16S rRNA regions were amplified using a two-step PCR protocol, first amplifying the gene region using universal primers 27F and 534R and then adding sample barcodes and sequence adapters. Amplicons were sequenced using the Illumina MiSeq at the University of Idaho. Forward and reverse reads were paired using FLASH (47) and processed through DADA2 v 1.12.1 (48) to identify distinct sequences, and the distinct sequences were classified to genus and species levels using SPINGO (49).

10.1128/mSphere.00943-20.1 TEXT S1 A description of methods used for the isolation of genomic DNA, production of 16S rRNA gene amplicons, DNA amplicon sequencing, and whole-genome shotgun sequencing. Download Text S1, DOCX file, 0.01 MB. Copyright © 2020 Nunn et al. 2020 Nunn et al. This content is distributed under the terms of the Creative Commons Attribution 4.0 International license.

For whole-genome shotgun sequencing, DNA libraries were prepared using the Nextera DNA library kit (Illumina) and pooled for sequencing at the IBEST Genomics Resources Core at the University of Idaho. Whole-genome shotgun sequencing was performed using the HiSeq 4000 at the University of Oregon. Sequences were quality trimmed and then filtered, assembled, and annotated using the metaWRAP pipeline (50). The resulting metagenomic assembled genomes (MAGs) were classified to the species level using gtdbtk (31).

### Proteomic analysis of vaginal fluids.

To determine the proteins present in vaginal fluids, CVM was processed using the MPLeX method, which was previously described (51, 52). Briefly, this method uses a solvent-based extraction that incorporates a mixture of water, chloroform, and methanol to extract proteins, lipids, and polar metabolites in three different fractions. The protein layer was placed into a separate tube at which point disulfide bonds of the proteins were reduced using 10 mM dithiothreitol for 1 h at 37°C, alkylated using 40 mM iodoacetamide at 37°C for 1 h, in the dark, and digested with trypsin for 3 h at 37°C. Salts and other nonpeptidic contaminants were removed by C_18_ solid-phase extraction before being analyzed by reverse-phase liquid chromatography coupled with tandem mass spectrometry (LC-MS/MS).

Five microliters of 0.1 μg/μl were analyzed by reverse-phase LC-MS/MS using a nanoEquity ultraperformance liquid chromatography (UPLC) system (Waters, Milford, MA, USA) coupled with a QExactive HF-X mass spectrometer (Thermo Fisher Scientific, San Jose, CA, USA). The LC was configured to load the sample first on a solid-phase extraction (SPE) column followed by separation on an analytical column, similarly to methods described previously (53). Briefly, the samples were separated using a 200-min gradient on a C_18_ in-house packed column. The effluents were analyzed with the Orbitrap mass spectrometer operated in the data-dependent analysis mode. The top 12 ions from the survey scan were selected for high-energy dissociation. An isolation window of 0.7 Da was used for the isolation of ions, and a collision energy of 28% was used for high-energy collisional dissociation (HCD) with an automatic gain control (AGC) setting of 3 × 10^6^ ions. The MS/MS scans were acquired at a resolution of 45,000. Mass spectra were recorded for 200 min by repeating this process with a dynamic exclusion of previously selected ions for 45 s.

### Statistics and proteomics data analysis.

All subsequent analyses were performed in R (version 3.6.0) (54). We used a generalized linear model with a log-link function to assess the relationship between amylase activity and glycogen levels. The pseudo-*R*^2^ for the model was calculated by subtracting the ratio of the model’s residual deviance and the null deviance from the value of one. Statistical significance for this model was set at a *P* value of 0.05.

To identify proteins in the four samples, the raw files generated were analyzed using MaxQuant (v1.6.6.0) (55) against a database comprising the proteins predicted from assembled metagenomes and the UniProt nonredundant human proteome. Proteins were quantified using label-free quantification (LFQ) and intensity-based absolute quantification (iBAQ) methods (56), which were performed using the match between runs, with a matching window frame of 1.5 min. Protein intensities were log_2_ transformed and median centered before computing the Z-scores for each protein using the package RomicsProcessor (https://doi.org/10.5281/zenodo.3386527) as previously described (57). Hierarchical clustering was performed using Ward’s method (58) on Euclidian distances calculated from normalized protein intensities.

### Data availability.

Raw sequencing data from 16S rRNA Illumina sequencing were deposited in the NCBI Sequence Read Archive (SRA) under BioProject accession number PRJNA628524. Whole-genome shotgun sequencing data were also deposited in the SRA under BioProject accession number PRJNA631294. The proteomics data generated were deposited on MassIVE (MSV000085378).
