# Supplementary material for: Amylases in the Human Vagina
Source: mSphere. 2020 Dec 9;5(6):e00943-20. doi: 10.1128/mSphere.00943-20 (PMC7729256; doi:10.1128/mSphere.00943-20)
Supplement: TABLE S2 [file mSphere.00943-20-st002.pdf]

Table S2. Relative proportions of bacteria in each community. These data were generated by analyzing count data from 16S rRNA gene amlicon sequencing.

	**Bacterial Taxa**										
Study Participants	*Lactobacillus crispatus*	*Lactobacillus iners*	*Lactobacillus jensenii*	*Gardnerella vaginalis*	*Atopobium vaginae*	*Lactobacillus gasseri*	*Megasphaera*	*Bifidobacterium pseudocatenulatum*	*Prevotella bivia*	Other	Total
F01	0.98	0.01	0.01	0	0	0	0	0	0	0	1
F02	0.02	0	0	0	0	0.98	0	0	0	0	1
F03	0	0.69	0	0.15	0.05	0	0.08	0	0	0.02	1
F04	0.66	0.3	0.01	0	0	0.01	0	0	0	0.02	1
F05	0.01	0	0.98	0	0	0	0	0	0	0	1
F06	0.05	0.95	0	0	0	0	0	0	0	0	1
F07	1	0	0	0	0	0	0	0	0	0	1
F08	0.84	0	0	0.15	0.01	0	0	0	0	0	1
F09	0	0	0.98	0.02	0	0	0	0	0	0	1
F10	0	0.98	0	0.01	0	0.01	0	0	0	0	1
F11	0.98	0.02	0	0	0	0	0	0	0	0	1
F12	0.99	0	0.01	0	0	0	0	0	0	0	1
F13	0	1	0	0	0	0	0	0	0	0	1
F14	0.43	0.55	0.02	0	0	0	0	0	0	0	1
F15	1	0	0	0	0	0	0	0	0	0	1
F16	0.99	0	0	0	0	0	0	0	0	0	1
F17	0.24	0	0.04	0	0	0	0	0.6	0.09	0.03	1
F18	0	0.96	0.04	0	0	0	0	0	0	0	1
F19	0	0.03	0	0.78	0.07	0	0.04	0	0	0.08	1
F20	1	0	0	0	0	0	0	0	0	0	1
F21	0	0.73	0.27	0	0	0	0	0	0	0	1
F22	1	0	0	0	0	0	0	0	0	0	1
F23	0.97	0.02	0.01	0	0	0	0	0	0	0	1
