# Supplementary material for: Amylases in the Human Vagina
Source: mSphere. 2020 Dec 9;5(6):e00943-20. doi: 10.1128/mSphere.00943-20 (PMC7729256; doi:10.1128/mSphere.00943-20)
Supplement: TABLE S3 [file mSphere.00943-20-st003.pdf]

Table S3. Attributes of CVM samples collected from reproductive age women.

	N	Mean	CI
Volume (µl)	23	469.6	383.4 - 555.8
pH	23	4.3	4.1 - 4.4
Total protein (mg/ml)	23	3.4	2.7 - 4
Amylase activity (U/mg protein)	23	0.6	0.3 - 0.8
Glycogen (mg/ml)	23	7.2	4.1 - 10.3
D-lactic acid (mM)	23	47.5	31.7 - 63.3
L-lactic acid (mM)	23	41.4	27.9 - 54.9
Total Lactic acid (mM)	23	88.9	66.6 - 111.1
