# Supplementary material for: Amylases in the Human Vagina
Source: mSphere. 2020 Dec 9;5(6):e00943-20. doi: 10.1128/mSphere.00943-20 (PMC7729256; doi:10.1128/mSphere.00943-20)
Supplement: TABLE S4 [file mSphere.00943-20-st004.pdf]

Table S4. Characteristics of samples selected for shotgun metagenomics and proteomic analysis

Parameter	Subject			
	F02	F06	F08	F12
pH	4.5	4.4	5	3.9
Total protein (mg/mL)	3.41	1.4	1.73	1.45
amylase activity (U/mg protein)[a]	0.06	1.32	0.61	0.36
glycogen (mg/mL)	13.9	15.7	7.27	32.75
D-lactic acid (mM)	38.31	0.63	16.16	119.82
L-lactic acid (mM)	26.03	35.11	10.73	45.64
Total Lactic acid (mM)	64.35	35.75	26.89	165.46
Dominant bacteria[b]	*Lactobacillus gasseri*	*Lactobacillus iners*	*Lactobacilus crispatus*	*Lactobacillus crispatus*

[a] amylase activity is expressed in units of amylase (U) per mg of total protein in the sample.

[b] dominant bacteria corresponds to bacteria that have a relative abundance ≥ 50% of the vaginal commun
