# Supplementary figures and images for: Amylases in the Human Vagina
Source: mSphere. 2020 Dec 9;5(6):e00943-20. doi: 10.1128/mSphere.00943-20 (PMC7729256; doi:10.1128/mSphere.00943-20)

(A)

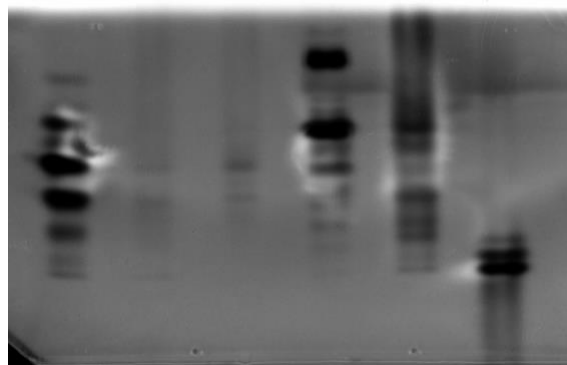

PA F01 F02 F03 F04 GA

(B)

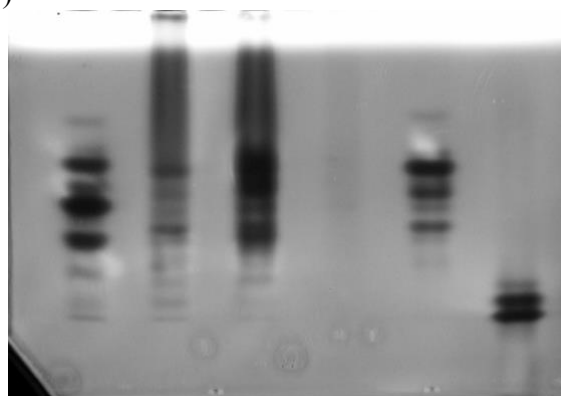

PA F05 F06 F07 F08 GA

(C)

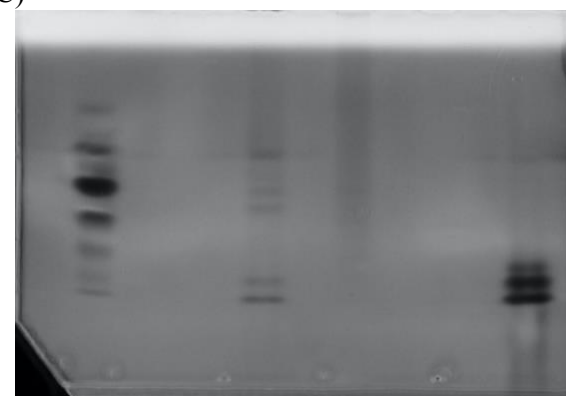

PA F09 F10 F11 F12 GA

(D)

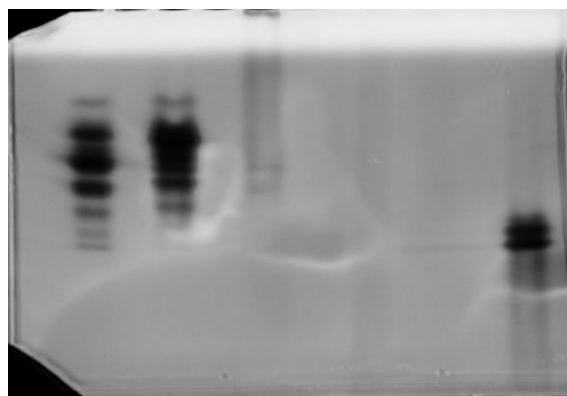

PA F13 F14 F15 F16 GA

(E)

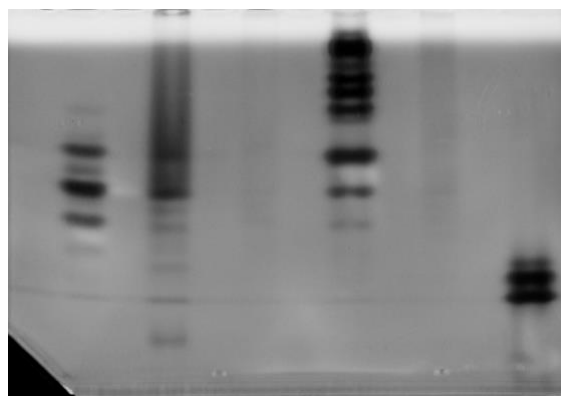

PA F17 F18 F19 F20 GA

(F)

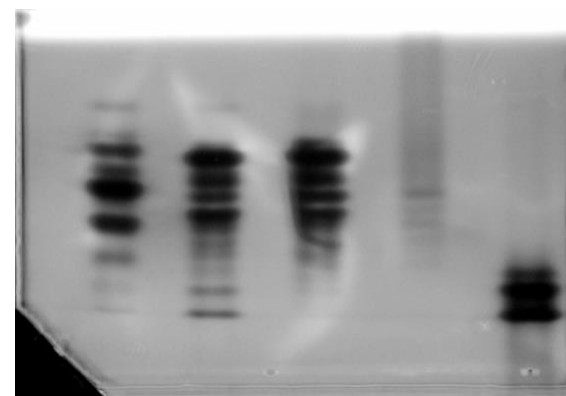

PA F21 F22 F23 GA

Supplement: FIG S1 [file mSphere.00943-20-sf001.pdf]

Euclidean distance

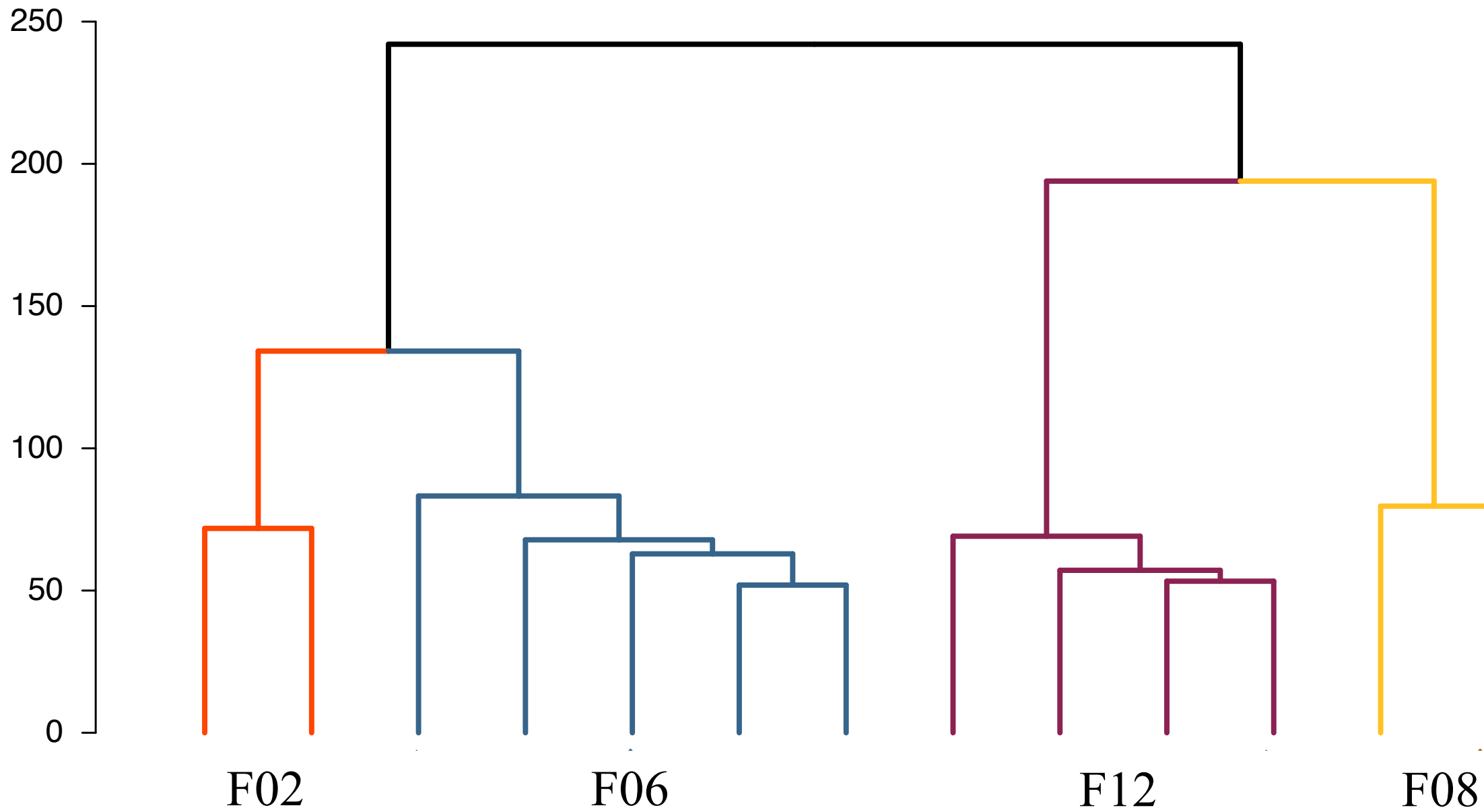

Supplement: FIG S2 [file mSphere.00943-20-sf002.pdf]
