# Supplementary material for: Amylases in the Human Vagina
Source: mSphere. 2020 Dec 9;5(6):e00943-20. doi: 10.1128/mSphere.00943-20 (PMC7729256; doi:10.1128/mSphere.00943-20)
Supplement: TEXT S1 [file mSphere.00943-20-s0001.docx]

# SUPPLEMENTAL TEXT

## Isolation of genomic DNA

The V1–V3 region of bacterial 16S rRNA genes (*Escherichia coli* positions 27F-534R) was amplified using a mixture of degenerate primers that flanked the variable regions. The sequences of these primers are shown here:

Table. Primers used for amplification of V1-V3 variable regions.

**NAME SEQUENCE**

534R_YM1-F ACACTGACGACATGGTTCTACAGTAGAGTTTGATCCTGGCTCAG

534R_YM2-F ACACTGACGACATGGTTCTACACGTAGAGTTTGATCATGGCTCAG

534R_YM3-F ACACTGACGACATGGTTCTACAACGTAGAGTTTGATTCTGGCTCAG

534R_YM4-F ACACTGACGACATGGTTCTACATACGTAGAGTTTGATTATGGCTCAG

534R_Bif-F ACACTGACGACATGGTTCTACAGTACGTAGGGTTCGATTCTGGCTCAG

534R_Bor-F ACACTGACGACATGGTTCTACACGTACGTAGAGTTTGATCCTGGCTTAG

534R_Chl-F ACACTGACGACATGGTTCTACAACGTACGTAGAATTTGATCTTGGTTCAG

27F_1-R TACGGTAGCAGAGACTTGGTCTCCATTACCGCGGCTGCTGG

27F_2-R TACGGTAGCAGAGACTTGGTCTGCCATTACCGCGGCTGCTGG

27F_3-R TACGGTAGCAGAGACTTGGTCTTGCCATTACCGCGGCTGCTGG

27F_4-R TACGGTAGCAGAGACTTGGTCTATGCCATTACCGCGGCTGCTGG

27F_5-R TACGGTAGCAGAGACTTGGTCTCATGCCATTACCGCGGCTGCTGG

27F_6-R TACGGTAGCAGAGACTTGGTCTTCATGCCATTACCGCGGCTGCTGG

27F_7-R TACGGTAGCAGAGACTTGGTCTATCATGCCATTACCGCGGCTGCTGG

## DNA amplicon sequencing

Equal amounts of amplicons (∼100 ng) were pooled in a single tube. The amplicon pool was cleaned to remove short undesirable fragments using the following procedure. First the pool was size selected with using AMPure beads (Beckman Coulter Inc., Pasadena, CA, United States), the product was then run on a 1% gel, excised from the gel, column purified using a Qiagen MinElute PCR purification kit and size selected again with AMPure beads (Beckman Coulter, Indianapolis, IN, United States). To determine the quality of the amplicons, the pool was PCR amplified with Illumina adaptor specific primers followed by size selection using a DNA1000 chip and an Agilent 2100 Bioanalyzer. The cleaned amplicon pool was then quantified using the KAPA Illumina library quantification kit (KAPA Biosciences) and the Applied Biosystems StepOne plus real-time PCR system. Finally, sequences were obtained using an Illumina MiSeq paired-end 300 bp protocol (Illumina, Inc., San Diego, CA, United States). Amplicon sequencing was performed in the IBEST Genomic Resources Core at the University of Idaho.

**Whole-genome shotgun sequencing**

DNA libraries were prepped using the Nextera DNA library kit (Illumina) and pooled at the IBEST Genomics Resources Core at the University of Idaho. Libraries were quantified using the Applied Biosystems StepOne plus real-time PCR system, analyzed for fragment size distributions using the AATI Fragment Analyzer (Agilent), and sequenced using the HiSeq 4000 at the University of Oregon.
